# Specific educational strategies using the Anatomage table for physical and occupational therapy students: a questionnaire-based survey

**DOI:** 10.3389/fmed.2025.1615890

**Published:** 2025-11-12

**Authors:** Akira Yoshikawa, Ryo Takagi, Megumi Enokida

**Affiliations:** 1 Division of Morphology, Anatomy and Physiology, Department of Medical Basics, Specialty and Education, Graduate School of Nursing and Rehabilitation Sciences, Showa Medical University, Yokohama, Kanagawa, Japan; 2 Division of Health Science Education, School of Nursing and Rehabilitation Sciences, Showa Medical University, Yokohama, Kanagawa, Japan; 3 Department of Physiology, School of Medicine, Showa Medical University, Tokyo, Japan; 4 Division of Physical Therapy, Department of Rehabilitation, School of Nursing and Rehabilitation Sciences, Showa Medical University, Yokohama, Kanagawa, Japan; 5 Division of Health Science Education, Department of Medical Basics, Specialty and Education, Graduate School of Nursing and Rehabilitation Sciences, Showa Medical University, Yokohama, Kanagawa, Japan; 6 Department of Nursing, School of Nursing and Rehabilitation Sciences, Showa Medical University, Yokohama, Kanagawa, Japan

**Keywords:** Anatomage table, rehabilitation education, educational strategies, digital educational tool, anatomy, physiology, physical therapy, occupational therapy

## Abstract

**Introduction:**

Anatomy and physiology are important basic medical sciences and foundational subjects in rehabilitation courses. As an educational tool, the Anatomage table, a virtual dissection table, can help medical and allied health science students to improve their academic motivation and gain a deeper three-dimensional (3D) understanding of human anatomy, to train them as future healthcare professionals. However, specific educational strategies using this tool are unclear. This study therefore aimed to identify specific educational strategies for students enrolled in rehabilitation courses using the Anatomage table, guided by Kolb’s Experiential Learning Cycle (ELC).

**Methods:**

This study involved conducting six neurophysiology lectures, after which the participating physical and occupational therapy students attempted two tasks (report and quiz assignments), specifically designed for this study. After completing the assignments, the students provided the necessary feedback by completing a questionnaire. Qualitative content analysis and correspondence analysis were subsequently conducted.

**Results:**

Our findings revealed that the assignments left a positive impression on many students, enhanced their understating of the human body’s 3D structure, and promoted peer collaboration. The report assignment, which was associated with “Concrete Experience” and “Reflective Observation” in the ELC, was generally perceived as more engaging than the quiz. However, it also revealed that students found both assignments difficult without the required prior knowledge or preparation. The clear instructional videos and stable system performance improved usability, whereas scheduling constraints and limited foundational knowledge were perceived as challenges.

**Discussion:**

These findings suggest that the assignments conducted using this tool support the visual learning preferences typical of early-year rehabilitation students. The sequential use of report and quiz tasks may enhance experiential learning by linking reflection and active experimentation. Integrating such technology-enhanced strategies within a pedagogical framework can suggest complement traditional lectures and cadaver-based anatomy education.

## 1 Introduction

Anatomy and physiology are important basic medical sciences and essential foundational subjects in rehabilitation courses, including physical and occupational therapy (1). One of the challenges facing the instructional approach or strategy in anatomy and physiology is the promotion of active learning using current technologies. Medical education currently employs technologies, such as computer-assisted learning, mobile learning, digital games, simulations, and wearable technologies (2). Anatomy lectures are conducted using three-dimensional (3D) tools, augmented reality, virtual reality, web-based programs, tablet-based applications, etc., (3), while physiology lectures are conducted using game elements (4). In the last decade, the Anatomage table, a virtual dissection table, has been used as a basic medical science learning technological tool for digital learning in anatomy and 3D learning in physiology, educating medical students and those taking medicine-related courses. Several reports highlighting the use the Anatomage tables in educational practices have observed increased motivation to learn; better understanding of the 3D structure between each organ; and the opportunity for students to experience simulated surgery using their fingers as scalpels, boosting their motivation as future medical professionals (5–7). While the Anatomage table improves 3D comprehension, specific educational strategies to optimize its use remain unclear.

To develop innovative educational strategies that leverage emerging technologies, it is essential to ground the discussion within a clear pedagogical framework. Such a theoretical foundation provides valuable insights for educators involved in medical and allied health science education. Various learning theories have previously been proposed as guiding frameworks in health profession education (8). For example, the Anatomage table enables learners to engage in concrete experiences by performing virtual dissections (e.g., removing the pericardium to expose the myocardium) and manipulating physiological functions (e.g., altering the heart rate) in a simulated environment. These concrete experiences encourage reflective observation, allowing students to consolidate or deepen the anatomical and physiological knowledge acquired through traditional, text-based classroom learning. Building on this reflection, learners engage in abstract experimentation by integrating structural and functional understandings—for example, recognizing how action potentials conducted through specialized cardiac muscle lead to the contraction of ordinary myocardial fibers. Ultimately, this process, which involves concrete experience (feeling), reflective observation (watching), abstract conceptualization (thinking), and active experimentation (doing), promotes active experimentation, in which learners independently consider how disruptions in normal structure and function may result in pathological conditions. This sequential process exemplifies Kolb’s Experiential Learning Cycle (ELC) (8), providing a theoretical basis for the educational use of the Anatomage table in anatomy and physiology instruction.

This study aimed to explore rehabilitation students’ perceptions of specific educational strategies using the Anatomage table based on Kolb’s ELC. We hypothesized that these strategies would be perceived as useful to encourage active learning (concrete experience) and enhancing students’ understanding of 3D anatomical structures (reflective observation). We designed the following nervous system-based tasks using the Anatomage table as part of a lecture course entitled “Neurophysiology (Basic)” (Supplementary Table 1). Task 1 involved students operating the Anatomage table to display the target structures appropriately, which is followed by them taking a screenshot of the displayed image, pasting it into a text file, and writing a report. Task 2 required students to answer a quiz in the Anatomage table. This study investigates specific educational strategies using the Anatomage table in neurophysiology lectures as it is an important area of study for students intending to become physical and occupational therapists, and the nervous system education is one of the current trends in this field (9).

## 2 Materials and methods

### 2.1 Participants

This study was conducted among second-year physical (*n* = 35: 21 females, 14 males) and occupational (*n* = 11: 8 females, 3 males) therapy students attending a neurophysiology (basic) lesson. Participants’ ages ranged from 19 to 21 years. All participants had successfully completed the foundational Anatomy and Physiology coursework (excluding the nervous system) in their first year. These prerequisite courses were traditionally delivered as didactic lectures using textbooks in a classroom setting, and none of the participants had prior experience with cadaveric dissection (gross anatomy laboratory experience). Thus, all second-year students used the Anatomage table version 10 (Anatomage, Inc., Santa Clara, CA, United States) for the first time in this course to visualize the structure and function of the human body in 3D. This study was reviewed and approved by the Ethics Committee of Showa Medical University (approval number: 2024-217-A). Students were informed about the study and consent was obtained through and opt-out process.

### 2.2 Task for students using the Anatomage table

The neurophysiology lesson consisted of six lectures conducted from April to July 2024 (Supplementary Table 1). Each lecture lasted 90 min, and students had to complete their assignments using the Anatomage table. There were two assignments: a report that had to be written using the illustration captured on the Anatomage table (Supplementary Table 1: Classes 1 and 6), and a quiz contained in the Anatomage table (Supplementary Table 1: Class 4 and 5). The reporting tasks included distinguishing between the central and peripheral nervous systems, projecting the corticospinal tract, and locating memory-related areas. Conversely, the quiz task was associated with hearing and visual functions and involved localization of the basal ganglia. Students tapped what they believed to be the correct structure on the screen in response to questions set by the instructor, and the system provided immediate feedback indicating whether the tapped structure was correct or incorrect. Approximately 10 questions were created for each session focusing on hearing, vision, and the basal ganglia. Students could review their overall performance at the end via a PDF file, which showed how many seconds it took them to answer each question and whether their answers were correct/incorrect. This allowed students to assess their own level of understanding through the quiz tasks. The scores for this quiz task were not included in the analysis, as the study aim was to explore the learning strategies used throughout the quiz tasks. The purpose of the assignments and a video manual explaining the Anatomage table were provided to students through the web-based learning platform, Google Classroom. Students could access this explanation video anytime and anywhere, and address the assignments. The deadline for each assignment was 2 weeks after completing the lectures. To complete these assignments, students used the Anatomage table.

### 2.3 Qualitative content analysis

A questionnaire survey was used to investigate the perceived benefits and challenges of learning the structure and function of the nervous system using the Anatomage table (Supplementary Table 2). The questionnaire was developed specifically for this exploratory study of physical therapy and occupational therapy students. Participants completed the questionnaire after completing all lectures and Anatomage-based assignments. Prior to responding, participants were informed that the purpose of the survey was to investigate how assignments completed using the Anatomage table contributed to students’ acquisition of knowledge. Anonymity of responses was assured to allay concerns about potential grade disadvantage. Students rated items using a four-point scale (very easy, easy, difficult, very difficult) and were also asked to provide free-text comments in response to the following: (1). How easy or difficult was it to operate the Anatomage table system? (2). How easy or difficult were the assignments, (3). Which was easier, the report or quiz assignment? and (4). Please provide your opinions on the assignments completed using the Anatomage table. Google Forms was used to administer the survey. Considering that this study involves a qualitative investigation, we applied the narrative synthesis method, providing a comprehensive understanding of complex topics, affording insights that are not confined by the rigid frameworks seen in other types of knowledge syntheses. Qualitative data, particularly free-text comments, were assessed for rigor.

Following our previous methodology (10) and that of Thornton et al. (11), Zheng et al. (12), qualitative content analysis was conducted through the following systematic procedure: Researcher A first compiled all student comments into an anonymized Excel file. Three researchers (A, B, and C)—one expert in medical education, one anatomist, and one physiologist—then independently and repeatedly reviewed the text data to identify any meaningful themes. The identified themes were subsequently organized into clusters of similar content, and categorized as positive or negative opinions. After independent coding, the three researchers met to discuss and reconcile any discrepancies, refining overlapping codes and reaching a consensus on the final categories. Redundant information was minimized, and the core meanings of the responses were clarified through iterative discussion. To supplement the qualitative findings and explore linguistic patterns, we additionally conducted correspondence analysis using KH Coder (Version 3. Beta.04f), a text-mining tool utilizing artificial intelligence algorithms (13). Specifically, responses to Questions 1/2 were analyzed using correspondence analysis based on the previous report to identify characteristic groupings of words associated with each response category (“very easy,” “easy,” “difficult,” and “very difficult”) (14). Moreover, although Question 4 did not include such predefined categories, the free-text comments were analyzed to extract and classify distinctive clusters of words. This supplemental computational analysis served to reinforce and validate the thematic structures identified through qualitative content analysis. To ensure the reliability and credibility of the analysis, triangulation across the three researchers with distinct disciplinary backgrounds was conducted. One researcher involved in this process had prior experience in qualitative research, contributing to methodological rigor. Finally, the finalized Japanese text was translated into English and collectively reviewed to ensure accuracy and consistency.

## 3 Results

### 3.1 Assignment results using the Anatomage table

**FIGURE 1:**
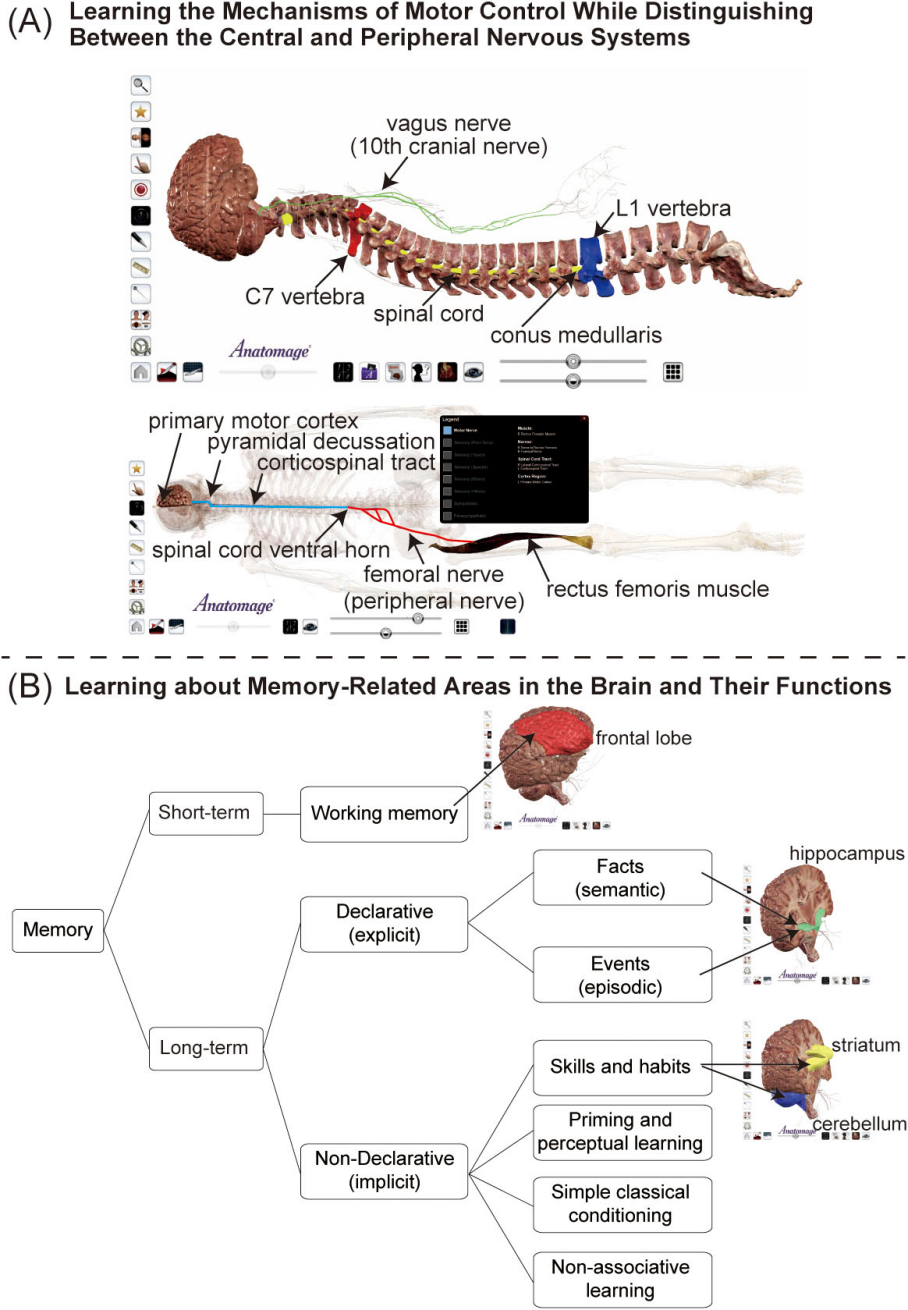
The report assignment. [**(A)**: upper] Classification of the central and peripheral nervous systems. Students were asked to create a diagram similar to that shown in the figure. They were asked to show the brain and spinal cord as the central nervous system, and the vagus nerve (the 10th cranial nerve) as the peripheral nervous system. To make it easier for students to understand, the spinal cord was coded in yellow and the vagus nerve in green. In addition, the 7th cervical vertebra (C7) was shown in red and the 1st lumbar vertebra (L1) in blue, for easy understanding of the spine levels, length of the caudal side of the spinal cord, and location of the vagus nerve endings. [**(A)**: lower] The content helped students to understand the corticospinal tract projection, which is a descending conduction pathway. Students were asked to visualize the projection starting from the primary motor cortex right up to the anterior horn of the spinal cord through the pyramidal decussation. In the figure, the corticospinal tract is in light blue, and after the synaptic connection at the anterior horn of the spinal cord, the projection from the peripheral nerves to the skeletal muscles continues to be seen. Additionally, the femoral nerve (peripheral nerve) innervating the right rectus femoris muscle is shown in red. **(B)** Areas responsible for human memory. Memory can be divided into short- and long-term. We focused only on working, declarative, and non-declarative memories, as these are associated with the skills and habits learned in the basic course. Students were asked to figure out their responsible regions anatomically.

After the lectures, students completed the assignment using the Anatomage table. There were two main assignments: a report and a quiz. In the first, students wrote about three themes. The first theme involved the distinction between the central and peripheral nervous systems (Figure 1A: upper). Displaying the brain and spinal cord and column in their anatomical locations helps students understand their locations. In particular, characterizing the lumbar level of the spinal cones, and subsequently the cauda equina, leads to learning application in the field of orthopedic surgery. Using the Anatomage table, students were asked to classify the central and peripheral nervous systems. In the Anatomage table, the cranial nerves classified as peripheral nerves, which often confuse students, were displayed. Specifically, the vagus nerve (the tenth cranial nerve) was used as an example. This assignment helped students understand the categorization of the central and peripheral nervous systems (Figure 1A: upper). The second theme involved understanding the projection of the corticospinal tract, for example, the descending projection pathway of the right rectus femoris. After making students aware that the corticospinal tract originates from the pyramidal cells in the left primary motor cortex, they were asked to recognize how it descends down the right spinal cord during pyramidal decussation in the medulla oblongata. They were asked to construct a display indicating that the corticospinal tract synapsed to the femoral nerve, a peripheral nerve in the anterior horn of the lumbar spinal cord, and femoral nerve projected a nerve to the right rectus femoris muscle. The students’ construction of this display helped them understand the connections from the central nervous system to peripheral nerves during movement and peripheral nerves to effector organs (skeletal muscles) (Figure 1A: lower). The third theme was to learn about the various types of memory and the responsible brain regions. After the students learned about short- and long-term memory and the characteristics of each memory type, they were asked to write a report on the relationship between memory and the brain’s regions of responsibility. In particular, students were asked to focus on parts of the long-term memory, such as the frontal lobe, hippocampus, and striatum and cerebellum, responsible for the working, fact and event, and skill and habit memories, respectively (Figure 1B).

**FIGURE 2:**
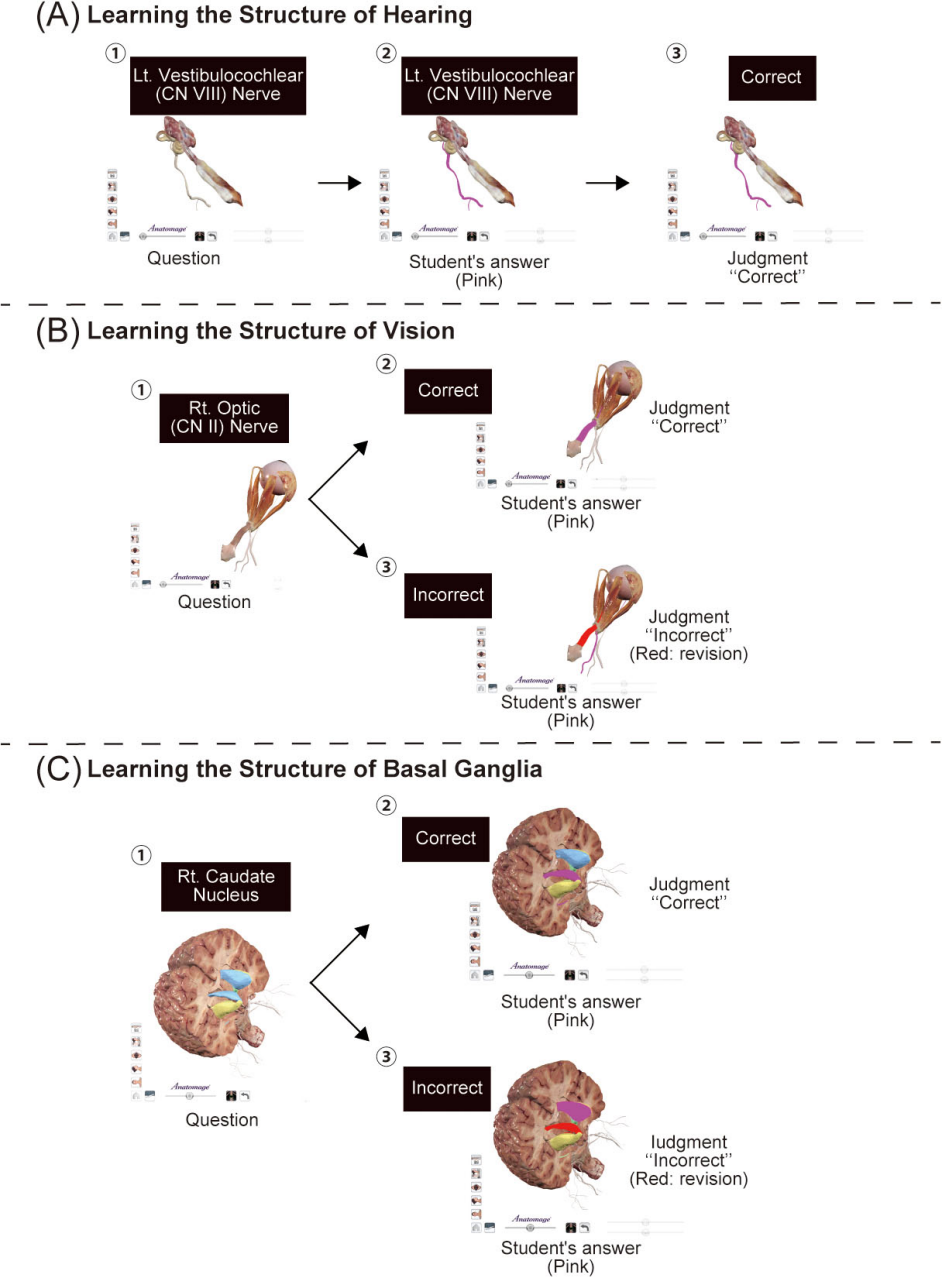
The quiz assignment. **(A)** Overview of the quiz on the structure and functions of the ear. The example shown in the figure illustrates a student’s answer to a quiz on the inner ear nerve. To answer the question indicated by the black square in panel **(A)**-①, the student taps on the anatomical table, reflected in pink in panel **(A)**-②. If the answer is correct, it is indicated as a correct answer, as seen in panel **(A)**-③. **(B)** Overview of the quiz on the structure and functions of the eye. The example shown in panel **(B)**-① is a student’s answer to a quiz on the optic nerve. If the structure tapped by the student (pink) is correct, the message “judgment correct” is displayed, as seen in panel **(B)**-②; however, if incorrect, the correct structure is shown in red, as seen in panel **(B)**-③. **(C)** An example quiz task on the structure and function of the basal ganglia. In panel **(C)**-①, we see a question on the right caudate nucleus. To provide a hint, each region of the basal ganglia is indicated in light blue, yellow, and green. If the structure tapped by the student (pink) is correct, the message “judgment correct” is displayed, as seen in panel **(C)**-②; however, if incorrect, the correct structure is shown in red, as seen in panel **(C)**-③.

The second assignment involved answering a quiz in the Anatomage table application. The quiz contained questions about vision and hearing structure and functions, as well as the classification of the basal ganglia. The Anatomage table application allows faculty members to create quiz questions. In this study, participants were asked, “Which is the cochlear nerve?,” related to the hearing structure and function (Figure 2A). On tapping the appropriate option, the selected structure appeared in pink, along with a message saying, “judgment correct,” indicating that the right option has been tapped. This was followed by a question on vision, “Which is the optic nerve?” (Figure 2B). Selection of the correct and incorrect structures led to a pink message “judgment correct” and a red message “judgment incorrect,” respectively. Similarly, Figure 2C shows an example of the basal ganglia classification, involving the anatomical locations of the caudate nucleus, putamen, and globus pallidus, which students find complex. Combined, the caudate nucleus and putamen and putamen and globus pallidus are called the striatum and nucleus lenticularis, respectively, which is complicated for students to understand. Therefore, students were asked to perform the task in a quiz format. Reports containing the results of the quiz, comprising the answers, percentage of correct answers, and response time, were submitted by the participating students to the faculty.

### 3.2 Questionnaire results

Responses were obtained from all 46 students who attended the neurophysiology lecture (Table 1). Most students’ responded “very easy or easy” to Question 1. However, approximately 20% found the system “difficult to use.” The free-text responses from students can be summarized as follows: (i) Operability and instructional support: Students reported that the table was easy to operate when clear instructional materials were provided during lectures; however, students experienced difficulties in operating the system when these materials were inadequate. (ii) Visual impact for learning human structures: The large display supported student understanding of anatomical structures, offering strong visual support in 3D; nonetheless, occasional technical malfunctions disrupted the learning process. (iii) Technical and system performance constraints: Once students became familiar with the system, its use was generally well-accepted. However, when the system performance was unstable or slow, it interfered with students’ learning processes. In response to Question 2, 50% of the students answered, “very easy or easy,” and the remaining, “difficult or very difficult.” The free-text responses expanded upon the themes identified in Question 1, namely, “operability” and “the value of 3D visualization in understanding human anatomy.” Two distinctive themes emerged: peer collaboration and students’ level of foundational knowledge acquisition. The responses indicated that students actively supported each other through peer learning while engaging in assignments, thereby promoting active learning. Conversely, students noted difficulties related to scheduling and reserving time to use a single Anatomage table collaboratively. Moreover, many students reflected that successful assignment completion required sufficient foundational knowledge. Those who had adequately reviewed and prepared in advance found the assignments relatively easy, whereas those with limited prior study perceived them as more difficult. The answers to Question 3 revealed that 56.5% of students preferred the report assignment, whereas 43.5% preferred the quiz. The free-text responses to Question 4, which provided overall reflections on the use of the Anatomage table, yielded four overarching themes: (i) the tool enables a three-dimensional understanding of human anatomy; (ii) it allows students to enjoy collaborative learning experiences with peers using a novel educational tool; (iii) it functions as an effective means for retaining, confirming, and outputting acquired knowledge, and (iv) it requires the effective implementation of careful instructional design and operational planning in educational settings.

**TABLE 1:** Results of the questionnaire survey.

Question 1: How easy or difficult was it to operate the Anatomage table system?			
–	Very easy	26.1%	–
–	Easy	52.2%	–
–	Difficult	21.7%	–
–	Very difficult	0%	–
**Theme**	**Summary of the relevant opinions**	**Positive statements**	**Negative statements**
Ease of operation and learning support	Instructor-provided materials contributed to mastering the operation.	“The explanatory video provided by the lecturer made it easy to operate.” “Because the explanation using the video was appropriate.”	“It was difficult to operate without watching the video.” “There were many buttons, and I was confused at first.”
Visualization and understanding of anatomical structures	Supported spatial understanding and learning effect with 3D display function.	“We could look at the anatomical structures in 3D and understood where several structures were in each others.” “The large display made it easy to visualize muscles and nerves because the body structure could be viewed in 360 degrees”	“The rotation control was difficult to handle.” “The response was slow when trying to change the viewing angle.”
Technical and system performance constraints	Concerns about the complexity of the initial operation (delays during startup and operation) and the need to get used to it.	“Once we got used to it, the operation was smooth.”	“It took some time to start up.” “The operations were slow, so sometimes we were concerned about whether it was working or not.”
**Question 2: How easy or difficult was the assignment?**			
–	Very easy	4.3%	–
–	Easy	45.7%	–
–	Difficult	43.5%	–
–	Very difficult	6.5%	–
**Theme**	**Summary of the relevant opinion**	**Positive statements**	**Negative statements**
Peer collaboration	Cooperation with friends was the main factor reducing the difficulty of the assignment.	“Because we were able to work together with everyone.” “It felt easy because we were able to cooperate with our peers.”	“It was difficult to coordinate the schedule.” “We need to make a booking to use this machine.”
Need for prior knowledge/preparation	The difficulty of the assignment stems not from the tools, but from a lack of knowledge due to the lecture content.	“Because it was something I could answer based on the lecture content and textbook.” “I could have understood it if I had researched it myself before the assignment. However, I found using the Anatomy Table made it easier to understand.”	“Because I did not have enough knowledge.” “Because my knowledge was not completely fixed yet.” “It was difficult if we didn’t understand the content of the lectures.”
**Question 3: Which was easier, the report or quiz assignment?**			
–	Report assignment	56.5%	–
–	Quiz assignment	43.5%	–
**Question 4: using the free text cell, please provide your opinion on the assignments using the Anatomage table.**			
**Theme**	**Summary of the relevant opinion**	**Positive statements**	**Negative statements**
Learning effectiveness through 3D visualization	Supported spatial understanding and realistic learning experiences via 3D display capabilities.	“I thought it was incredibly realistic and easy to understand.” “Being able to see everything in 3D deepened my understanding.” “It was good to be able to see details that we usually can’t see.”	−
Enjoyment and motivation with new learning technology	Cooperation with friends and using new technology enhanced enjoyment and motivation for learning.	“It was fun because I followed a new studying method.” “It was easy to understand because we could cooperate with friends while having fun.”	−
Knowledge retention and output	Assignment formats (quizzes, visual operations) were supported for knowledge verification and memorization.	“It was a good opportunity to output what I learned in class, and my understanding deepened.” “Since we can learn visually, it is easier to remember.” “I was able to deepen my knowledge through the test on the Anatomage table.” “I was able to move and check each organ by myself, so I understood better.”	“Quizzes were difficult for me.”
Technical and operational challenges	Initial operational difficulty and requests regarding the system (e.g., we need to book or use only one Anatomage table).	−	“Booking it was a hassle.” “I know it is very expensive, but I felt like we needed one more unit.” “The operation was difficult for me”
Pedagogical support and instruction	Instructor-provided materials contributed to skill acquisition and assignments clarification.	“The assignment was very easy to follow since the lecturer explained it in a video.”	–

**FIGURE 3:**
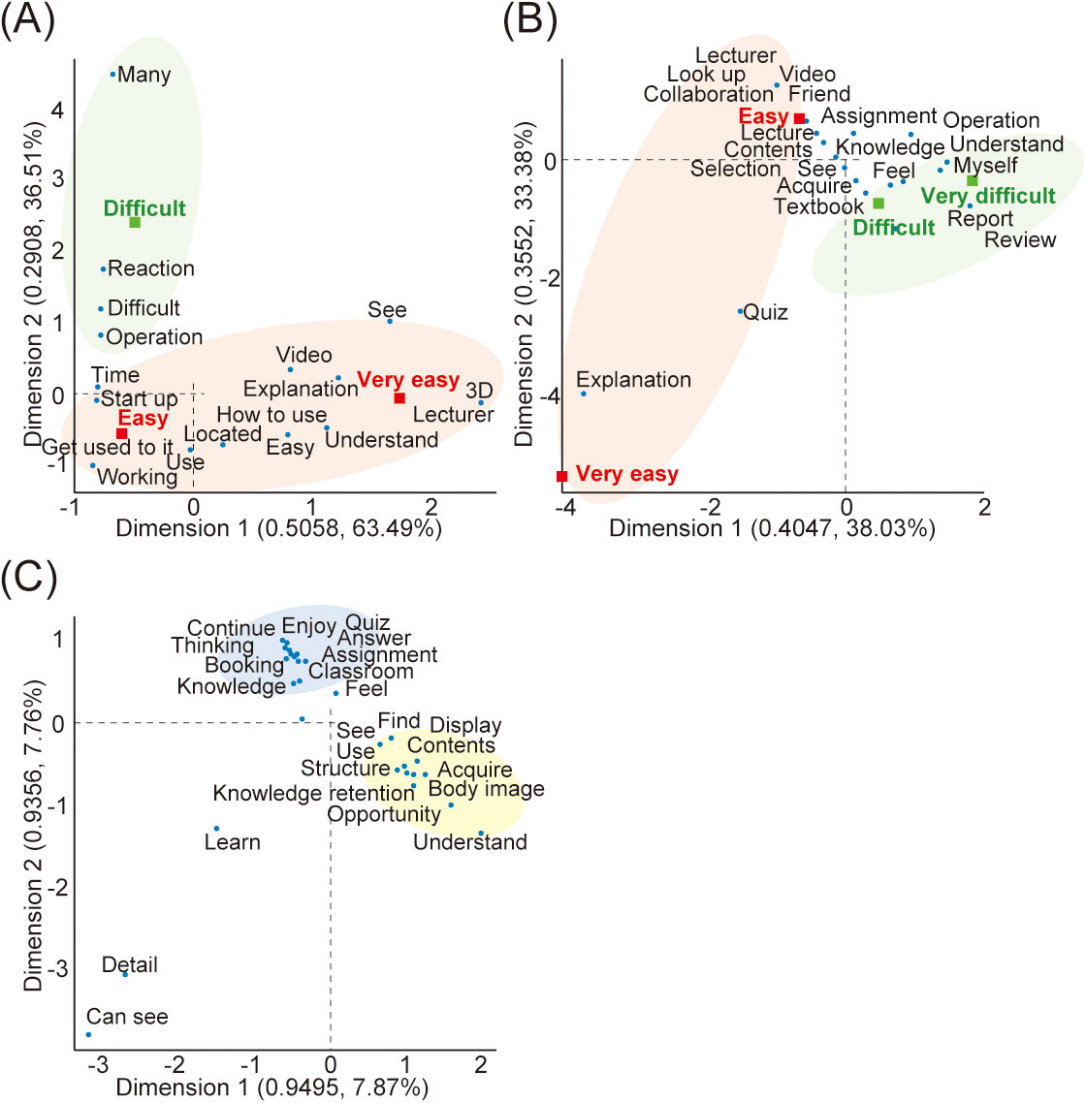
Results of the correspondence analysis of the students’ free-text responses using KH Coder. Correspondence analysis was conducted to visualize the characteristic word clusters from students’ comments for each question. Each plot shows that the farther the distance from the origin (0,0) along the x and y axes, the more distinguishable and distinctive the word clusters become. The area outlined in red indicates the words characteristic of “Very easy and Easy,” the area outlined in green indicates word groups related to “Difficult and Very difficult,” the area outlined in yellow indicates word groups related to knowledge and understanding gained through learning, and the area outlined in blue indicates word groups related to internal emotions and hardware aspects during the learning process. **(A)** For Question 1, terms such as “3D,” “Lecture,” “Explanation,” and “Video” were associated with “very easy/easy,” whereas “Many” and “Reaction” were linked to “difficult,” reflecting operational complexity. **(B)** For Question 2, “Collaboration” and “Friend” characterized “easy” responses, suggesting peer-supported learning, while terms related to insufficient knowledge appeared in “difficult” clusters. **(C)** For Question 4, clusters including “Body image,” “Structure,” “Knowledge retention,” and “Acquire” highlighted perceived educational benefits, whereas “Booking” indicated logistical challenges in using the Anatomage table.

To further explore the textual characteristics summarized in Table 1, we conducted correspondence analysis using KH Coder (Figure 3). Figures 3A–C present the results of the correspondence analysis for the free-text comments from Questions 1/2/4, respectively. In each plot, the greater the distance from the origin (0,0) along the x- and y-axes, the more distinct the word clusters became. In Question 1 (Figure 3A), the words “3D,” “Lecture,” “Explanation,” and “Video” were closely associated with the categories “very easy” and “easy,” suggesting that instructional videos and clear explanations provided by the lecturer contributed to improved usability. In contrast, the cluster for “difficult” contained the words “Many” and “Reaction,” indicating that students perceived the large number of control buttons and occasional system lag as sources of operational complexity. In Question 2 (Figure 3B), the words “Collaboration” and “Friend” were characteristic of the “very easy” and “easy” responses, implying that peer interaction and mutual support facilitated learning. Conversely, clusters representing “difficult” and “very difficult” were associated with terms suggesting challenges arising from insufficient prior knowledge and the need for additional review during the report-writing process. In Question 4 (Figure 3C), clusters containing the words “Body image,” “Structure,” “Knowledge retention,” and “Acquire” indicated that students recognized the Anatomage table as an effective tool for understanding body structures and reinforcing learned knowledge. Another cluster included the terms “Enjoy,” “Assignment,” “Quiz,” and “Answer,” reflecting enjoyment and engagement through collaborative peer learning. However, the appearance of the word “Booking” within the same cluster highlighted a logistical issue—specifically, the difficulty of scheduling and reserving a single available Anatomage table for group use.

## 4 Discussion

This study explored the advantages and disadvantages of implementing specific educational strategies using an Anatomage table based on Kolb’s ELC that allows users to actively learn the human body structures in 3D. The findings promote new possibilities for technology-enhanced lectures and practical anatomy and physiology sessions.

Participants stated that they were able to understand the spatial relationships of structures in 3D through assignments using the Anatomage table. The report assignment required students to virtually dissect specific structures and describe the functions of the dissected nerves based on their own generated images. According to Kolb’s ELC, this report task could be positioned within the stages of “Concrete Experience” and “Reflective Observation.” Conversely, the quiz task required students to respond to questions based on previously learned content, primarily corresponding to “Reflective Observation,” as it allows learners to consolidate/deepen their understanding (8). When comparing the two learning strategies, students generally preferred the report assignment. The report assignment involves both “Concrete Experience” and “Reflective Observation,” which have been associated with a “Diverging” learning style often preferred by first-year medical students (15). Furthermore, a study investigating learning style preferences among healthcare professional students reported that visual and auditory learning styles were favored in the early years of medical education (16). Together, these results suggest that the report assignment, which enabled students to visually engage in both “Concrete Experience” and “Reflective Observation,” was preferred over the quiz assignment. However, it is important to note that students were required to acquire relevant knowledge in advance to effectively engage with the tasks in both types of assignments. Students who possessed sufficient prior knowledge appeared to enjoy using the visualized educational tool in both assignments, whereas those with limited knowledge experienced difficulty. These findings highlight the importance of preparation and self-directed pre-study to promote effective engagement and deeper understanding, not only within the study context, but also more broadly in learning.

Another key element identified for successfully implementing both assignments with new technology was the provision of explicit instruction, to help students become comfortable with the technology and to clarify each task’s learning objectives. Although many students are digitally native, some may still struggle with unfamiliar tools (17). For such students, providing carefully designed instructional videos may have helped reduce anxiety towards the technology. In addition, explicitly stating the learning goals appears to be fundamental. Previous work suggests a positive relationship between clearly-defined learning goals and learners’ self-efficacy (18). In our study, the instructional video included both practical operation guidance and a concise explanation of the educational purpose for each assignment. Together, these measures likely made the tasks more transparent, increased motivation, and facilitated enjoyable, collaborative engagement among peers.

The Anatomage table has affective elements, such as motivating students to learn and uplifting their morale about their future as healthcare professionals (19, 20). herein, students—particularly those without direct faculty guidance—actively engaged in discussions with their classmates as part of active learning during the assignments (Table 1 and Figure 3), supporting our earlier interpretation that the report assignment reflected a “Diverging” learning style, which encourages discussion and multiple perspectives, thereby promoting active peer collaboration among students (15). In contrast, the quiz assignment corresponded to a learning mode characterized by “Reflective Observation,” in which students receive feedback based on their own responses. When students possessed sufficient prior knowledge, they perceived the quiz positively, describing it as an opportunity for “self-assessment” and “deepening understanding” (Table 1 and Figure 3). However, when students attempted the quiz with insufficient background knowledge, they reported experiencing “difficulty” and a heightened awareness of their own knowledge gaps. Herein, the report and quiz assignments were presented as independent tasks rather than sequential components of a single learning process. Therefore, the outcomes may have differed if the quiz was designed to build upon the report assignment. These findings suggest that further refinement is required to design the report and quiz tasks in a manner that fosters a synergistic effect, thereby enhancing students’ overall learning experience.

While technology-driven medical education is important, many students feel that practical anatomy experience using cadavers is necessary (17, 21). Faculty believe that medical education using technological innovations should complement traditional anatomy lectures and practical anatomy sessions using cadavers (22, 23). Agreeing with the above-mentioned studies’ findings, we suggest a hybrid strategy combining both the traditional textbook-lecture method and practical training using cadavers and the new technology-driven educational style using the Anatomage table to achieve a synergistic effect in education. This can be achieved through the provision of clear learning themes, objectives, and curriculum planning (17, 24). Considering these findings, the primary educational implication of this study is that report/quiz assignments that utilize visual information can serve as an effective instructional strategy for early-year students. The use of the Anatomage table in these assignments may stimulate several key aspects of active learning: enabling students to visualize human anatomy in 3D, allowing repeated exploration, and fostering enjoyable peer collaboration (25). Engaging in such experiences during the early stages of medical education may gradually facilitate the transition towards a “Converging” learning style, encompassing both “Abstract Conceptualization” and “Active Experimentation,” as described in Kolb’s ELC. This developmental shift is considered crucial for promoting understanding of pathological alterations in normal structures and functions, as well as for supporting advanced clinical education in rehabilitation therapy and program design.

### 4.1 Limitations and future directions

This study examined the potential of report and quiz assignments using the Anatomage table as concrete educational strategies, based on a questionnaire survey. Although the questionnaire items were carefully developed and refined by three investigators, formal psychometric validation was not conducted; therefore, the results should be interpreted as derived from a pilot tool. Moreover, this investigation focused primarily on students’ learning strategies, and whether these strategies contribute to improvements in academic performance remains to be determined. In addition, the present study was conducted at a single institution and included only students majoring in physical and occupational therapy. Future studies should expand to multiple institutions and include students from other medical courses, such as medicine and nursing. Finally, previous reports have indicated that certain organ systems are easier to learn with the Anatomage table than others (26). As this study focused on the nervous system, further research should examine whether the effectiveness of these learning strategies varies depending on the anatomical theme.

### 4.2 Conclusion

This study investigated specific educational strategies to learn the nervous system in physical and occupational therapy courses using the Anatomage table, guided by Kolb’s ELC. The findings indicate that tasks emphasizing “Concrete Experience” and “Reflective Observation”—particularly report-based assignments—enhanced students’ engagement, motivation, and understanding of 3D anatomical structures. Conversely, quiz-based tasks facilitated knowledge consolidation and self-assessment, but were perceived as more challenging by students with limited prior preparation. These observations suggest that sequentially combining report/quiz tasks may strengthen experiential learning by linking reflection with “Active Experimentation.” Educationally, the Anatomage table supports visual and kinesthetic learning preferences common in early-year rehabilitation students, promoting active and collaborative learning. When properly designed, such technology-enhanced strategies can complement traditional lecture-based and cadaveric anatomy education, bridging theoretical knowledge with practical understanding. Future curriculum development should adopt hybrid approaches integrating emerging digital tools with experiential learning principles to enhance knowledge retention, clinical reasoning, and learner autonomy in rehabilitation education.

## Data Availability

The original contributions presented in this study are included in this article/Supplementary material, further inquiries can be directed to the corresponding author.
