# Supplementary material for: Specific educational strategies using the Anatomage table for physical and occupational therapy students: a questionnaire-based survey
Source: Front Med (Lausanne). 2025 Nov 12;12:1615890. doi: 10.3389/fmed.2025.1615890 (PMC12646991; doi:10.3389/fmed.2025.1615890)
Supplement: Supplementary file 1 [file Data_Sheet_1.pdf]

Supplementary Material

1 Supplementary Figures and Tables

1.1 Supplementary Tables

Supplementary Table 1. Overview of the curriculum and assignments designed for the study

**Course Name**	Neurophysiology (basic)		
**Target students**	2nd year (physical and occupational therapy)		
**Course Aim**	To introduce the basics of neurophysiology to help students to understand the fundamental principles of clinical neurology and neurorehabilitation.		
**Class**	**Course content**	**Course objectives**	**Assignment by using the Anatomage table**
1	- Basics of central nervous system physiology<br>- Structure of the central nervous system<br>- Brainstem reflexes<br>- Neural pathway	1. To list the parts of the central nervous system.<br>2. To list the parts of the cerebral cortex.<br>3. To explain the functional localization of the cerebral cortex.<br>4. To list the types of reflexes controlled by the brainstem.<br>5. To explain the mechanisms of brainstem-controlled reflexes by type.<br>6. To explain the types and characteristics of neural activity-based brain waves observed from the cerebral cortex.<br>7. To list the conduction pathways.<br>8. To explain the conduction pathways.<br>9. To explain the roles of the conduction pathways.	• Visually distinguish between the central and peripheral nervous systems using the Anatomage table.<br>• Project the corticospinal tract using the Anatomage table.<br>• Figure 1**(A)**

2	- Function of the frontal lobe<br>- Function of the parietal lobe<br>- Function of the temporal lobe<br>- Function of the occipital lobe	10. To explain the structure and functions of the frontal lobe.<br>11. To explain the structure and functions of the parietal lobe.<br>12. To explain the structure and functions of the temporal lobe.<br>13. To explain the structure and functions of the occipital lobe.	Anatomage table not to be used. Students use a checklist on Google Forms to provide the answers.
3	- Types and functions of somatosensory sensations	14. To list the types of somatosensory sensations.<br>15. To list the receptors of somatosensory sensations.<br>16. To explain the functions of the somatosensory receptors.<br>17. To explain the conduction pathways of somatosensory sensations.	Anatomage table not to be used. Students use a checklist on Google Forms to provide the answers.
4	- Types and functions of the special senses (vision, hearing, balance, smell, and taste)	18. To list the types of special senses.<br>19. To explain the functional localization of the special senses.<br>20. To explain the conduction pathways of the special senses.	• Using the quiz mode in the Anatomage table (answer questions related to the eye anatomy and visual function).<br>• Using the quiz mode in the Anatomage table (answer questions related to the ear anatomy and hearing function).<br>• Figure 2**(A)** and (**B)**
5	- Functions of the thalamus<br>- Functions of the basal ganglia<br>- Functions of the cerebellum	21. To explain the structure and functions of the thalamus.<br>22. To explain the structure and functions of the basal ganglia.<br>23. To explain the structure and functions of the cerebellum.	• Using the quiz mode in the Anatomage table (to answer questions related to the location of the basal ganglia).<br>• Figure 2**(C)**

6	- Functional Localization of the language areas<br>- Brain regions responsible for memory<br>- Brain regions and processes involved in memory formation and learning	24. To explain the functional localization of the language areas.<br>25. To explain language disorders.<br>26. To list the brain regions responsible for memory.<br>27. To explain the process of memory formation.<br>28. To explain the types of memory.<br>29. To explain the process of memory learning.	• Display the location of the memory-related areas.<br>• Figure 1**(B)**

Supplementary Table 2. Original Questionnaire Items

Questionnaire on Learning with the Anatomage table

Instructions: You recently used the Anatomage table for an assignment in the Neurophysiology (Basic) lesson. This anonymous survey requests your honest feedback regarding this learning experience. Your candid input is highly valued. Your grade or academic standing will not be affected in any way by your decision to participate or not participate, or by your responses.

Demographic Data Collection

Please indicate your academic affiliation. [Select one.]

- ○ Physical therapy course
- ○ Occupational therapy course

Please answer Questions 1 to 4 below.

Question 1-1: How easy or difficult was it to operate the Anatomage table system? [Select one.]

- ○ Very easy
- ○ Easy
- ○ Difficult
- ○ Very difficult

Question 1-2: Please state the reason for your selection in Question 1-1.

[Open-ended Comments]

Question 2-1: How easy or difficult was the assignment? [Select one.]

- ○ Very easy
- ○ Easy
- ○ Difficult
- ○ Very difficult

Question 2-2: Please state the reason for your selection in Question 2-1.

[Open-ended Comments]

Question 3: Which was easier, the report or quiz assignment? [Select one.]

- ○ Report assignment
- ○ Quiz assignment

Question 4: Using the free text cell, please provide your opinion on the assignments using the Anatomage Table.

[Open-ended Comments]
